# Addressing inequitable access to hospice care

**DOI:** 10.1136/bmjspcare-2022-003590

**Published:** 2022-06-16

**Authors:** Catriona R Mayland, Sarah Mitchell, Kate Flemming, Lynn Tatnell, Lesley Roberts, John I MacArtney

**Affiliations:** 1 Department of Oncology and Metabolism, The University of Sheffield, Sheffield, UK; 2 Department of Health Sciences, University of York, York, UK; 3 Patient and Public Representative, Torbay, UK; 4 Patient and Public Representative, Stafford, UK; 5 Warwick Medical School, University of Warwick, Coventry, UK

**Keywords:** COVID-19, Hospice care, Home care

## Abstract

The ‘Lancet Commission on the Value of Death’ proposes radical change and challenges the very core of hospice service provision. Without action, inequalities in access to hospice care will continue to be amplified. The COVID-19 pandemic brought increased needs and demands in the community setting but also provided opportunities for new palliative partnerships and ways of working. Returning to the status quo should not be an option. Rather moving towards a shared vision and purpose, which has the person and their community network at its centre, enables hospices to have a pivotal role and bring about more equitable palliative care.

The recent ‘Lancet Commission on the Value of Death’ draws on lessons from around the world to propose radical changes to help us ‘reimagine how death and dying could be’.^1^ Globally, the COVID-19 pandemic has challenged the very core of how institutional hospice care should be provided. Additionally, it has raised specific challenges for countries such as the UK, where hospices are often small-scale charities, providing inpatient care as well as community-based services.^2^ In this editorial, we will outline factors that influenced the evolving changes and argue that:

1. Without action, the recognised inequalities in access to hospice care will continue to be amplified.
2. The increased palliative care needs and demands within the community setting provide an opportunity for new partnerships and ways of working to be cemented, particularly with primary care.
3. The knowledge, skills and leadership from hospice institutions will have a pivotal role in influencing and integrating future care.

Globally, it is estimated that only 14% of people in need of palliative care can access services.^3^ The COVID-19 pandemic has been described as a ‘perfect storm’, exposing marked health inequalities related to socioeconomic deprivation, poor housing and low income.^4^ This is a pressing issue across all aspects of health and social care, including for palliative and end-of-life care provided by hospices. Inequitable access to hospice care is a long-standing concern: those with a non-cancer diagnosis, living in a rural or more socioeconomically deprived area, identified as having an ethnic minority background or aged over 85 years are less likely to receive care from hospice services.^5 6^ Criticism about the provision of a high-quality service for the privileged few, should challenge us to consider the potential of institutional hospice care to address inequalities in the future. Indeed, without meaningful self-reflection and subsequent action, the risk of returning to the status quo is very real.

Since the start of the COVID-19 pandemic, patterns of mortality in places such the UK and Canada have undergone huge shifts.^7 8^ While the number of deaths occurring within in-patient hospice services in England, Wales and Northern Ireland reduced by 15%, deaths at home showed a sustained and continued increase of 41%.^6^ Internationally, specialist palliative care services reported the need to shift their focus to provide more care in the community.^8 9^ Community healthcare services have played a critical role in responding to the increased need and complexity of end-of-life care within both home and care home settings. This has not been without significant emotional impact and has compounded the workload pressure for doctors and nurses working within the community.^10^ The increased number of deaths occurring in the community, compared with inpatient hospice services, has also shifted the burden of care for families. Pre-pandemic, this was already a recognised issue within specific healthcare systems (eg, USA) where hospice care at home is financially driven and support for caregivers is limited.^11^

These factors should help us refocus on the future direction of hospice care. The issues relating to adequate funding and resourcing of hospice care remain fundamentally important. For example, with the proposed Health and Social Care Bill in the UK,^12^ public campaigns have seized the opportunity to advocate for all those who are dying in England to be able to access the care they want and need. To ensure more equitable care is realised across the country, there is a call for it to be a legal duty to ensure the appropriate focus and resources are directed. This line of thinking is important but this action alone will not tackle the issue of providing long-term sustainable models of care to address unmet needs and ongoing inequalities in care provision. Hospice care will continue to be a finite resource. There is, however, an impetus and a recognised moral duty to broaden the reach and increase the numbers of patients which hospices serve as well as contributing to the overall provision of seamless, person-centred care.

The pandemic provided opportunities for initiatives that had previously been discussed to become reality. Within certain areas of the UK, hospices and community colleagues were able to be brought together with central contact points and a more integrated approach to working.^13^ Reports showed different services amalgamated to form multiprofessional teams providing both personal care and specialist community care.^9^ Increased application of technology was used to facilitate multidisciplinary team meetings with a broader membership or to provide daily staff updates. Internationally, initiatives such as Project ECHO^14^ enabled shared learning and facilitated the transfer of knowledge and skills to the wider workforce.^15 16^ The Lancet Commission challenges us to go further—hospices, as part of specialist palliative care services, need to advocate for building community capacity in its broadest sense, sharing knowledge and skills, and bringing networks together.^1^

Time is of the essence. Within the UK, for example, there are ongoing changes to the structures within their national health system. Integrated care systems (ICSs), partnerships bringing together those who prioritise and fund care, with local councils and providers of care services across specific geographical locations, are set to become statutory in 2022. Regionally, strategic networks representing Palliative and End-of-life Care have a key role in collectively guiding and supporting ICSs in the integration and improvement of patient care, advocating for ‘joined up’ approaches.

In view of evolving changes, we would advocate the following recommendations:

1. Leadership: There needs to be a move away from seeing ‘hospice care’ as that solely provided within a structure of a building or institution and more focus given to outreach and engagement within communities. Hospices’ specialist skills remain of vital importance, but they need to use their standing to lead and influence policy-makers and create an environment for community partnership.
2. Encouraging new approaches to hospice care: new ways of working should be encouraged to both generate novel initiatives and facilitate the sharing of positive outcomes. This includes valuing informal dialogue with a range of partners and stakeholders, as well as experimenting with innovative approaches to service provision. Support from robust research is needed and should not only value the generalisability of findings but provide evidence about how community care and institutional hospices can better work together in their specific locales and circumstances.
3. Engagement of patients, public and civic society: the patient voice is integral to guide the potential codesign of services and how they are organised and provided. Hospices need to open-up conversations with the public so they can directly influence how hospice care is provided in their community; how hospice care is connected across much wider health and social care systems; and, how hospice care can better engage in meaningful partnerships across societies.

## Summary

The COVID-19 pandemic has exacerbated many pre-existing healthcare inequities but has also accelerated many long-awaited and much needed changes to enable the provision of more palliative care within the community setting. It is now time for institutions and facilities which provide hospice care to reflect on how those inequities grew around them, despite their better efforts. Hospices can use their social and moral standing to instigate a shift in the discussion about how ‘good’ deaths can be accessible to all in their communities. Additionally, hospices can recognise the role that they play in amplifying the voice of their community to others. Moving towards a shared vision and purpose, which has the person and their community network at its centre, can help bring about more equitable palliative care.
